# Exceptional Cases Demand Exceptional Personalized Solutions: The Next Level in Dental Rehabilitation

**DOI:** 10.3390/jpm14030294

**Published:** 2024-03-09

**Authors:** Nils-Claudius Gellrich, Philippe Korn, Philipp Jehn, Michael Neuhaus, Fritjof Lentge, Björn Rahlf

**Affiliations:** Department of Oral & Maxillofacial Surgery, Hannover Medical School, Carl-Neuberg-Str. 1, D-30625 Hannover, Germanyjehn.philipp@mh-hannover.de (P.J.); rahlf.bjoern@mh-hannover.de (B.R.)

**Keywords:** patient-specific subperiosteal implant, IPS implants^®^ preprosthetic, computer-assisted planning, CAD/CAM, selective laser melting, complex dental rehabilitation, digital workflow, rigid fixation

## Abstract

Defects and bone loss in the maxilla and mandible pose significant challenges for dental rehabilitation. This paper focuses on complex cases of bimaxillary dental rehabilitation, where traditional dental implant protocols were not feasible in at least one jaw. Four patients were examined conceptually, where conventional dental implant placement (n = 20) was combined in either the same or opposite jaw with a patient-specific subperiosteal implant (n = 5). This study evaluates aspects such as primary stability, prosthodontic restoration, complications, and soft tissue management over the observation period. None of the five patient-specific subperiosteal implants (IPS Implants^®^ Preprosthetic; KLS-Martin Group, Tuttlingen, Germany) experienced failure or showed any loosening of screws, with the longest observation period extending to 68 months. These implants were securely fixated away from the posts, without any biomechanical restrictions on loading from the time of insertion. Planning and manufacturing, including the initial suprastructure, followed a fully digital workflow. The number of screws required for multivector fixation ranged from 13 to 22. All dental implants placed remain functional, definitive prosthodontic restoration has been performed, and no stability loss or peri-implantitis has been observed. The IPS Implants^®^ Preprosthetic emerges as a valuable consideration when conventional implant dentistry protocols encounter limitations.

## 1. Introduction

Implant dentistry has become the standard approach for addressing even the most challenging cases of dental rehabilitation. However, the conventional concepts of dental implants may encounter limitations when faced with difficult hard- and soft tissue conditions, or when patients are unwilling to undergo prolonged or invasive rehabilitation protocols, especially after experiencing failure in single or multiple bone augmentation and dental implant surgeries. This challenge is particularly pronounced in oncology patients following maxillary or mandibular ablation, where oral function may be significantly compromised, exacerbated directly or indirectly by adjuvant therapies such as radiation, chemotherapy, or long-term sequelae like growth restriction or scarring [1].

Over the past two decades, advancements in combined prosthodontic and computer-assisted planning, along with improved drill guide designs for guided surgery, have significantly enhanced the use of conventional dental implants in severely compromised patients [2,3]. Nonetheless, challenging scenarios underscore the limitations of conventional dental implant protocols, as they necessitate adequate bone volume at the implantation site [4]. This limitation differs fundamentally from the approach offered by the IPS Implants^®^ Preprosthetic; in the maxilla/midface, previous bone reconstruction/augmentation is unnecessary, while in the mandible, bone union is required, but the geometry of the implantation site is inconsequential. The primary requirement lies in robust soft tissue reconstruction in both the maxilla and mandible.

To address these challenges, the authors developed a novel patient-specific subperiosteal implant technique (IPS Implants^®^ Preprosthetic) starting in 2015. This technique achieves primarily stable multivector fixation on reliable buttresses of the maxilla, midface, or mandible through a subperiosteal implant with a framework—a patient-specific 3D-designed footplate manufactured using Selective Laser Melting (SLM) technology from titanium–vanadium–aluminum alloy (titanium grade V). This framework carries perfectly parallel aligned posts with telescoping and rotationally stable abutments in a one-piece design. Unlike conventional implants, where bone fixation occurs near the transition of pillars into the oral cavity, this bone anchorage principle allows for distant bone fixation, providing primary stability without reliance on osseointegration.

Following the inception of this technique in 2015, the authors treated 94 patients over nine years, encountering an increasing number of cases where conventional dental implant protocols had previously failed or would have necessitated overly invasive treatments. In these cases, the IPS Implants^®^ Preprosthetic proved to be a crucial bail-out strategy rather than a competitive method for dental rehabilitation [5,6]. The following cases highlight customized subperiosteal implants as a personalized extension in modern dental rehabilitation for complex cases.

## 2. Clinical Cases

### 2.1. Case No. 1

#### 2.1.1. Patient Presentation

**Figure 1:**
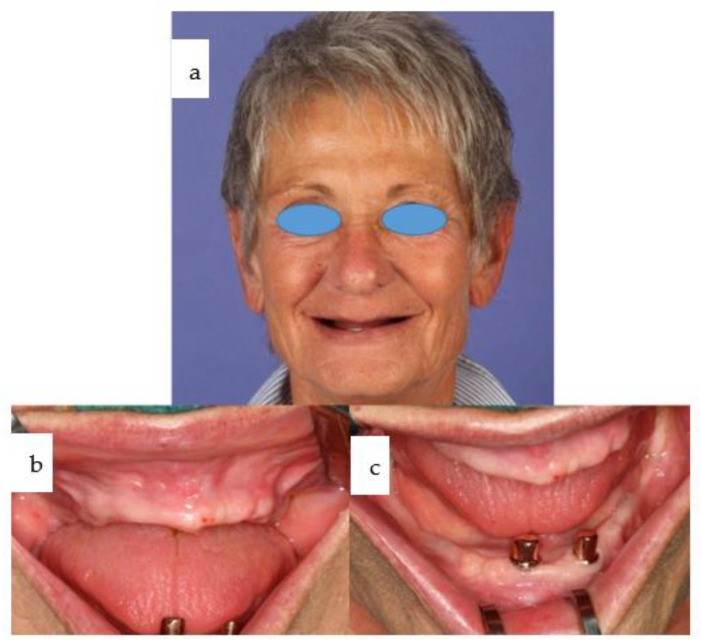
(**a**–**c**): En face view reveals deepened nasolabial folds and a typical pseudo-class III relationship resulting from severe maxillary atrophy. Intraorally, healthy but reduced tissues are observed in the edentulous maxilla and mandible. Two telescoping crowns are present on two out of three mandibular dental implants, which were previously placed elsewhere.

**Figure 2:**
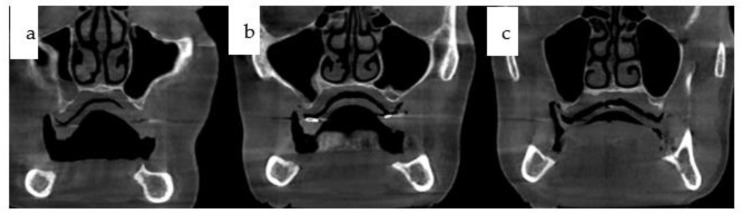
(**a**–**c**): Three cone-beam coronal views from the same patient in Figure 1 depict the reduced horizontal and vertical bone stock in the edentulous maxilla, displaying typical sequelae of atrophy-related centripetal bone loss.

A 66-year-old female patient presented with a non-functional overdenture in the maxilla, stabilized only by denture adhesive. In the lower jaw, a partially failed conventional implant-based dental rehabilitation was evident, with two out of three remaining dental implants functioning (Figure 1). Mandibular implants placed elsewhere exhibited deviations due to non-guided insertion, including vector misalignment in the coronal plane. One implant had to be retained as a sleeper due to its proximity to another implant in regio 33, while a fourth implant had already been lost without achieving secondary stability. Despite severe maxillary atrophy, soft tissue adequacy, combined with a high smile line, was fortunate (Figure 2).

#### 2.1.2. Treatment Challenges

A typical conventional treatment protocol involving lateral and vertical bone augmentation, including bilateral external sinus lifting, was deemed necessary for accommodating four to six conventional dental implants in the maxilla over a one-year treatment period. However, this approach was rejected by the patient. Previous dental implant treatment in the mandible had resulted in a significant skeletal pseudo-class III relationship, further complicated by attempts to align implant axes.

#### 2.1.3. Treatment Approach

**Figure 3:**
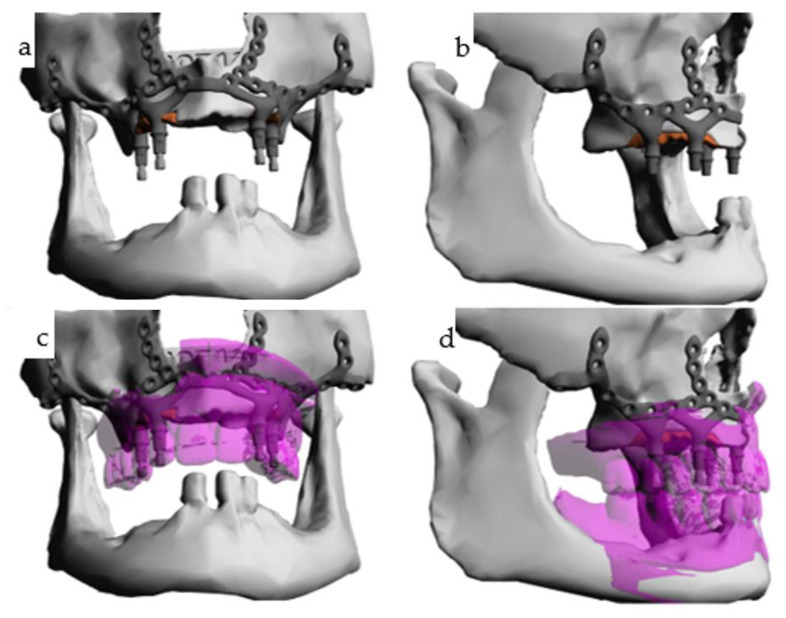
(**a**–**d**): Four screenshots from the case designer (KLS Martin-Group, Tuttlingen, Germany) display the one-piece subperiosteal implant (**a**). A red subvolume is delineated bilaterally in the atrophic maxilla, indicating a planned marginal bone resection (**a**–**d**). Four implant posts are deemed adequate to withstand occlusal forces in the edentulous maxilla (similar considerations apply to the mandible). Superimposition with the scanned pre-existing overdentures is depicted in (**c**,**d**), facilitating assessment of the implant-borne prosthodontic compensation for the skeletal pseudo-class III; this naturally requires intact intra- and extraoral soft tissues.

**Figure 4:**
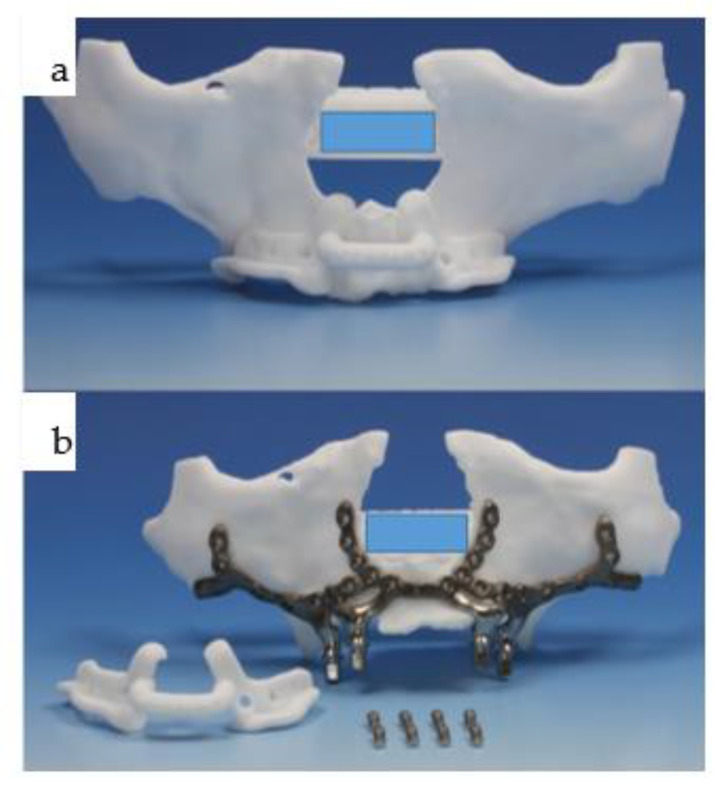
(**a**,**b**): The polyamide resection template is affixed to the patient’s biomodel in (**a**), featuring two extensions at the piriform aperture toward the nasal floor to ensure a one-fit-only design. Holes are provided for potential bony anchorage via screw fixation (note: due to the perfect fit, these holes have not been utilized by the authors thus far). The IPS Implants^®^ Preprosthetic (KLS Martin-Group, Tuttlingen, Germany) was then placed onto the biomodel, incorporating the intended bone resection in the maxilla, allowing the one-piece subperiosteal implant (**b**) to fit seamlessly into the bilateral marginal alveolar crest resection. In addition to the resection template, two sets of prosthodontic screws (four each) are visible: only four are necessary to secure the initial provisional suprastructure at the time of surgery and beyond, until the final prosthesis is fabricated.

**Figure 5:**
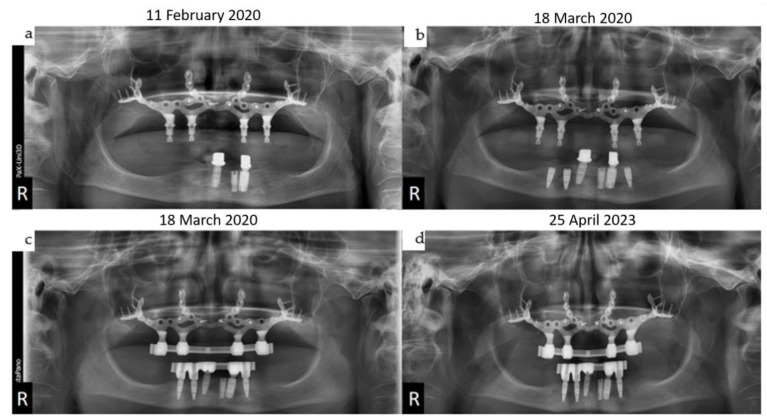
(**a**–**d**): Four orthopantomograms depict the dental rehabilitation sequence, commencing with the insertion of the IPS Implants^®^ Preprosthetic (KLS Martin-Group, Tuttlingen, Germany) (**a**); subsequently, the positions for the three bone-level tapered implants were drilled in a 3D-guided manner in a second step (**b**). During recall, the final bar-retained suprastructure (**c**) is visible, as well as at the three-year follow-up (**d**). No bone loss or screw loosening is evident. The key distinction between conventional implants and IPS Implants^®^ Preprosthetic anchorage is apparent: unlike conventional implants, where anchorage relies on multivector screw-based fixation near the area equivalent to the dental implant shoulder, the IPS Implants^®^ Preprosthetic constitutes a one-piece implant with a rotationally stable telescoping abutment component.

**Figure 6:**
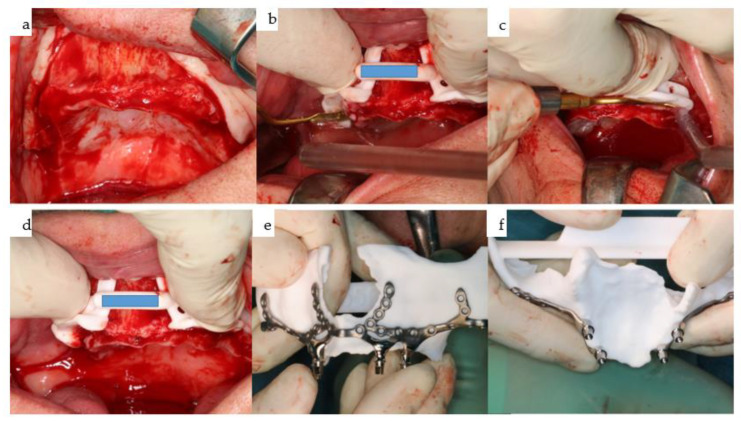
(**a**–**f**): Six intraoperative views illustrate the sequencing of IPS Implants^®^ Preprosthetic (KLS Martin-Group, Tuttlingen, Germany) insertion: first, the mucoperiosteal flap is harvested from the posterior right to left maxilla, with a small (1.2 cm) bilateral posterior vestibular periosteal release incision (**a**); next, piezosurgery is utilized for marginal bone resection on the right (**b**) and left (**c**) maxilla, employing the manually positioned and fixated resection template during (**b**,**c**) and after (**d**) the resection. Before insertion, a final check of the subperiosteal IPS Implants^®^ Preprosthetic is conducted on the autoclaved biomodel (**e**), revealing that the complex 3D design enables a one-fit-only design containing multiple holes for screw fixation, positioned far away from the transition zone through the soft tissues. The view from below (**f**) highlights one of the key features, whereby minimal metal is used around the transition through the soft tissues, while long booms extend onto the strongpoints of the maxilla and midface, utilizing the well-known medial and lateral midfacial buttresses for primarily rigid multivector fixation. (Annotation: The centric subnasal screw hole, which is typically present, allowing for the safe use of screw lengths up to 13 mm, is missing in this view.) The sloped design of the framework at each end of the extensions is noteworthy. Both the abutment part and the posts are highly polished, with the center of each post featuring a single prosthodontic screw thread design.

**Figure 7:**
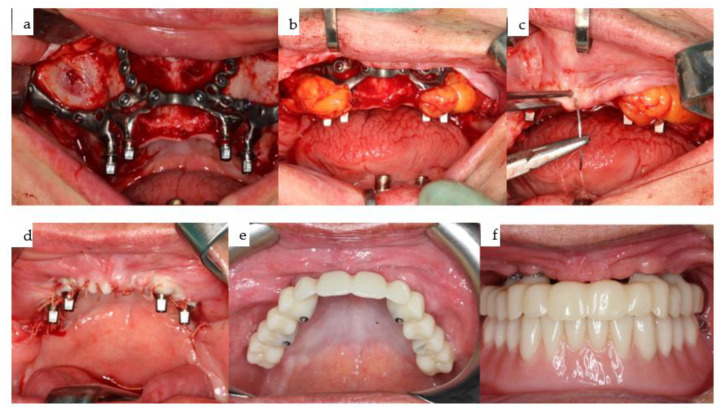
(**a**–**f**): Six clinical views illustrate the IPS Implants^®^ Preprosthetic (KLS Martin-Group, Tuttlingen, Germany) following multivector screw fixation with 22 1.5 mm screws in lengths ranging from 7 to 11 mm, prior to wound closure (**a**), after positioning the Bichat fat pad bilaterally around the lateral projection of the implant posts and securing them with 2.0 vicryl sutures (**b**), and at the onset of superficial wound closure following bilateral vestibular mucoperiosteal advancement flaps (**c**), culminating in tight wound closure. At six weeks postoperatively (sutures were removed three weeks postoperatively), the provisional suprastructure is visible in high-water design (with no soft tissue compression) (**e**–**f**).

To address the challenges, an IPS Implants^®^ Preprosthetic (KLS Martin Group, Tuttlingen, Germany) single-piece subperiosteal implant with four perfectly aligned posts for supporting a suprastructure was designed (Figure 3). This subperiosteal implant technique avoided additional bone grafting prior to implant placement. Focused marginal resection in the canine-to-second premolar regions of the maxilla was preplanned to accommodate the implant footplate (Figure 3, red subvolume). The CAD/CAM subperiosteal implant, manufactured using Selective Laser Melting (SLM) technology from titanium–vanadium–aluminum alloy powder, was inserted in February 2020 under general anesthesia during outpatient surgery (Figure 4, Figure 5, Figure 6 and Figure 7).

#### 2.1.4. Surgical Procedure

Primarily rigid fixation was achieved using 22 multivector 1.5 mm screws for the personalized subperiosteal implant (Figure 5). A provisional prosthesis, screw-retained and mounted onto the IPS Implants^®^ Preprosthetic, was immediately placed, allowing for unrestricted loading post-surgery.

#### 2.1.5. Follow-Up Procedures

**Figure 8:**
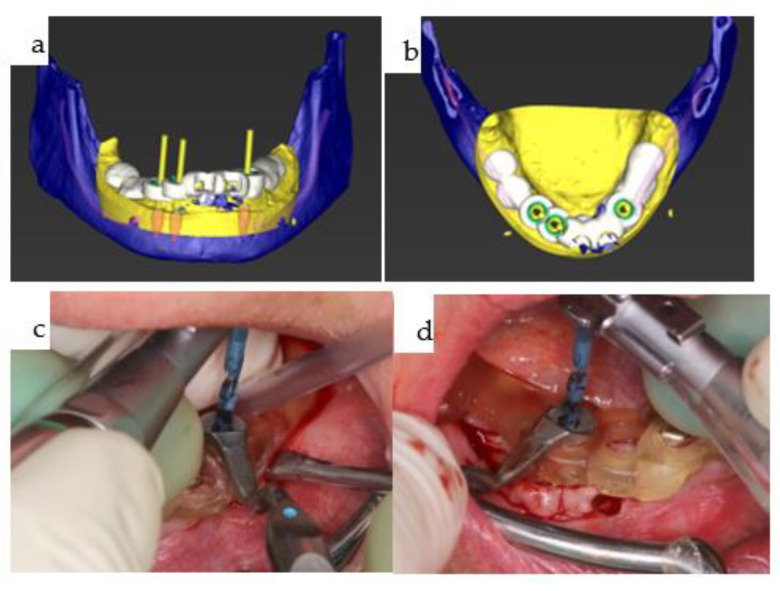
(**a**–**d**): Two screenshots from the coDiagnostiX^®^ dental implant planning platform (Straumann, Basel, Switzerland) depict the planned position of the three bone-level tapered implants and the design of the drill guide (**a**,**b**). The reduction spoon is utilized, with generic sleeves designed by narrowing down the diameter to match the inner metallic sleeve diameter, i.e., 5.0 mm (**c**,**d**). Guided drilling in the mandible is illustrated for both laterally placed dental implants (**c**,**d**).

Five weeks later, three bone-level tapered narrow-crossfit conventional dental implants were inserted using guided surgery (Figure 8). The drill guide, designed using coDiagnostiX 10.3 software (Straumann, Basel, Switzerland) and 3D-printed, facilitated precise placement. The conventional dental implants required uncovering three months later, while the maxillary IPS Implants^®^ Preprosthetic did not, as it already incorporated the abutment part.

#### 2.1.6. Prosthodontic Suprastructures

**Figure 9:**
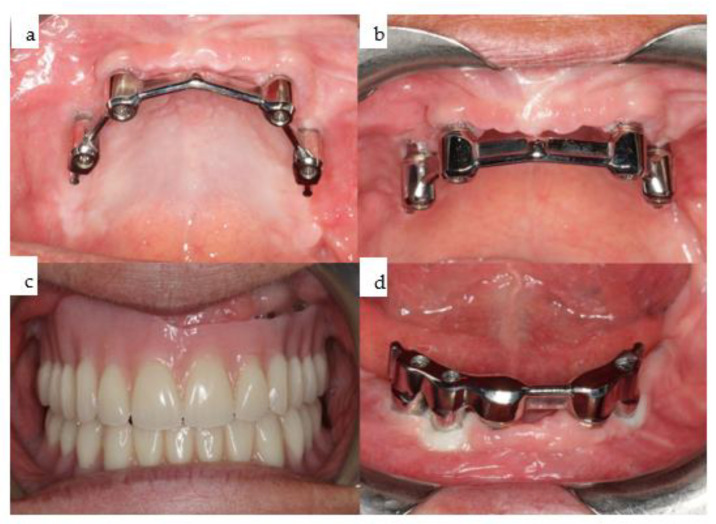
(**a**–**d**): Four intraoral views showcase the healthy tissues surrounding the four posts of the one-piece subperiosteal IPS Implants^®^ Preprosthetic (KLS Martin-Group, Tuttlingen, Germany). A bar-retained overdenture is utilized for both the maxilla and mandible (**a**–**c**), with the hard palate left uncovered. The maxillary bar is screw-retained on the four posts (**a**,**b**), while in the mandible, the bar is screw-retained on the three secondarily inserted implant abutments and telescoped onto the two previously placed implants placed elsewhere (**d**).

**Figure 10:**
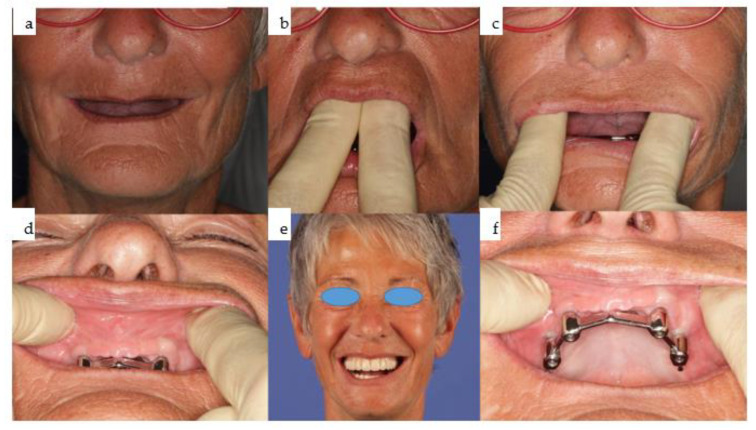
(**a**–**f**): Six clinical views illustrate the final outcome following bimaxillary dental rehabilitation, showcasing adequate separation of the vertical and horizontal subunits in the maxilla (**b**–**d**,**f**). An effective compensation for the skeletal pseudo-class III relationship is evident (**a**,**e**), with the overdenture design of the final prosthesis yielding the most cosmetically pleasing result, especially considering the high smile line (**e**).

The final prosthodontic suprastructures were designed as removable bar-retained overdentures, contributing to enhanced hygiene management and supporting lip and cheek projection (Figure 9 and Figure 10).

#### 2.1.7. Outcome

Four years post-treatment, the dental rehabilitation concept remains fully functional, with no evidence of peri-implantitis or mucositis observed during annual recall appointments.

### 2.2. Case No. 2

#### 2.2.1. Patient Presentation

**Figure 11:**
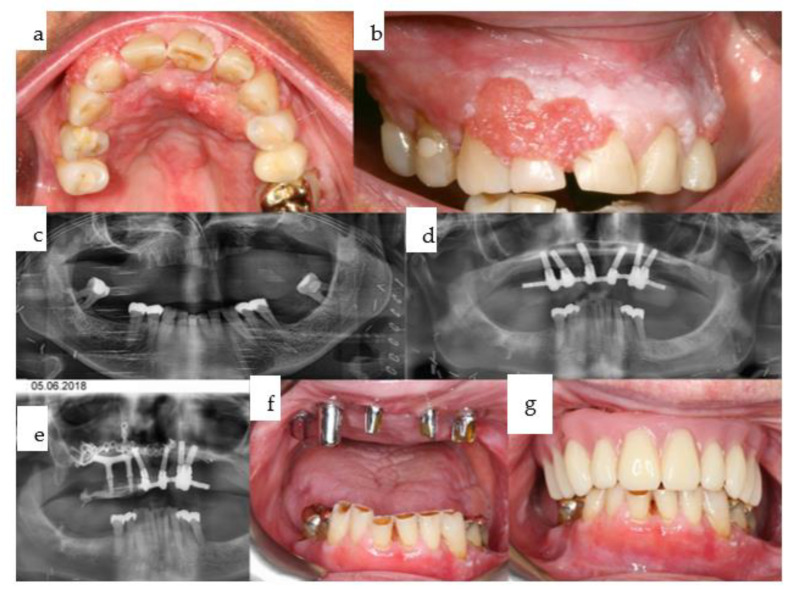
(**a**–**g**): Primary onset of squamous cell carcinoma of the maxilla began in 2012 following prolonged treatment with tacrolimus ointment administered by dermatologists (**a**,**b**), necessitating subtotal maxillectomy (**c**) with soft tissue reconstruction to separate the bilateral nasal and paranasal regions from the oral cavity. Dental rehabilitation was carried out using a conventional dental implant treatment protocol with a telescoping suprastructure (**d**). In June 2018, a right maxillectomy was required due to tumor recurrence, and a two-stage subperiosteal IPS Implants^®^ Preprosthetic procedure was performed (**e**), which was combined with bar-retained support along with the remaining 4 telescoping abutments on top of the dental implants (**f**). The prosthesis used was a removable overdenture designed to provide separation towards the vertical units of the cheeks and lips (**g**).

In 2012, a 70-year-old female patient developed squamous cell carcinoma of the maxilla after long-term treatment with tacrolimus ointment, which had been topically applied to the oral mucosa as therapy by dermatologists (Figure 11). Bilateral alveolar resection of the maxilla, including all teeth, was performed, followed by primary reconstruction using a microvascular lateral upper arm free flap to separate the vertical (lip, cheek, vestibule) from the horizontal subunits (Figure 11). Six conventional dental implants were subsequently guided and placed for the construction of a hard-palate-free-designed overdenture.

#### 2.2.2. Treatment Complications

Five years later, a tumor recurrence in the right maxilla necessitated extended segmental maxillectomy, including full hard palate resection. Bony ablation involved the removal of two posterior implants in the right maxilla.

#### 2.2.3. Treatment Approach

Following final histopathology confirming R0 resection, a delayed primary reconstruction was performed using a prelaminated latissimus dorsi myocutaneous free flap to achieve separation of subunits (free skin grafts harvested bilaterally from upper eyelids were used for prelaminating the latissimus dorsi muscle at the time of tumor resection). This two-stage surgical protocol is typically employed by the authors in cases of maxillary cancer. In addition to reestablishing the horizontal and vertical subunits, the free flap also served to separate the oral from the nasal and paranasal cavities. Six months after tumor resection, the patient opted against bone grafting of the right maxilla and midface, but expressed a desire for dental rehabilitation. Subsequently, a patient-specific subperiosteal implant was designed and manufactured using Selective Laser Melting (SLM) technology (Figure 11). The digital implant design included sufficient holes for screw fixation, though not every hole required mounting with a screw. Rigid fixation was achieved with multivector screws ranging from 7 to 13 mm in length, and the one-piece subperiosteal implant (titanium grade V) was prosthodontically planned and placed transorally in an outpatient procedure under general anesthesia.

#### 2.2.4. Prosthodontic Suprastructures

Initially, a provisional prosthesis was manufactured in a high-water design, followed by a final prosthetic suprastructure: a hard palate-free overdenture onto a bar (IPS Implants^®^ Preprosthetic) and four telescoping abutments (dental implants) (Figure 11).

#### 2.2.5. Outcome

**Figure 12:**
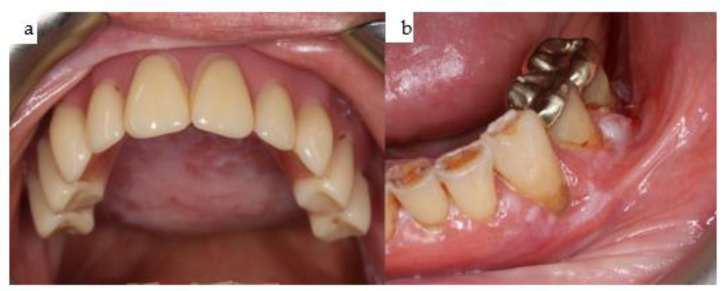
(**a**,**b**): In 2022, a secondary squamous cell carcinoma developed in the left mandibular canine to second premolar region (**b**). Maxillary form and function are fully compensated for with the removable hard-palate free overdenture in place (**a**).

For over 5.5 years, the dental rehabilitation concept has been fully functional, combining conventional dental implants with a secondarily placed patient-specific rigidly fixated subperiosteal implant, allowing retention of the main aspect of the original implant-borne concept (i.e., four dental implants) and full functional reconstruction of the ablated area without bone augmentation using an IPS Implants^®^ Preprosthetic. In 2022, the patient developed another squamous cell carcinoma in the left premolar gingival region of the mandible (Figure 12), necessitating further treatments and reconstructions.

#### 2.2.6. Subsequent Treatments

**Figure 13:**
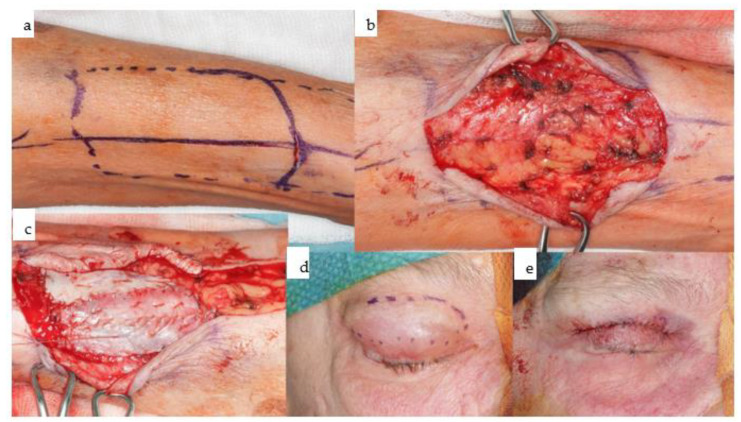
(**a**–**e**): Instead of employing a fasciocutaneous radial forearm flap directly 2–3 weeks prior to tumor resection, the preplanned donor site of the left radial forearm (**a**) is dissected subcutaneously (**b**) and prelaminated with full-thickness skin grafts (**c**). The donor sites for the skin grafts were both upper eyelids (**d**,**e**), although 5 years prior, this donor site had already been used to prelaminate the left latissimus dorsi flap.

**Figure 14:**
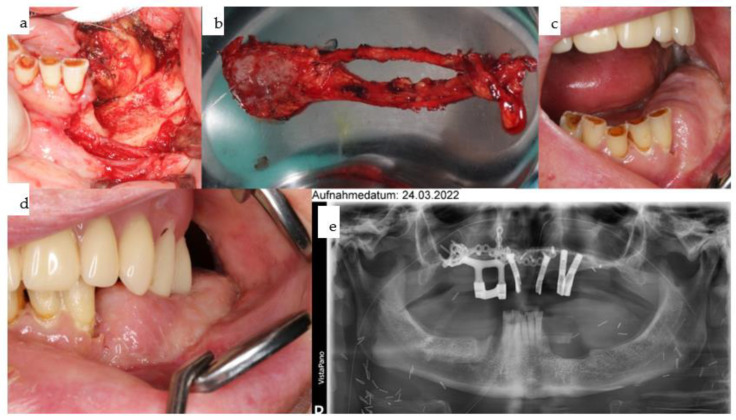
(**a**–**e**): Intraoperative view after tumor ablation due to mucosal cancer around the left canine-to-second-premolar region with marginal resection of the mandible (**a**); the prelaminated left radial forearm is harvested together with the cephalic vein prior to microvascular anastomosis (**b**). Twelve weeks post-resection and reconstruction, the prelaminated radial forearm flap shows a stable epithelialized surface (**c**,**d**). The orthopantomogram shows the marginal resection with numerous titanium microclips used for hemostasis during previous surgeries (**e**).

**Figure 15:**
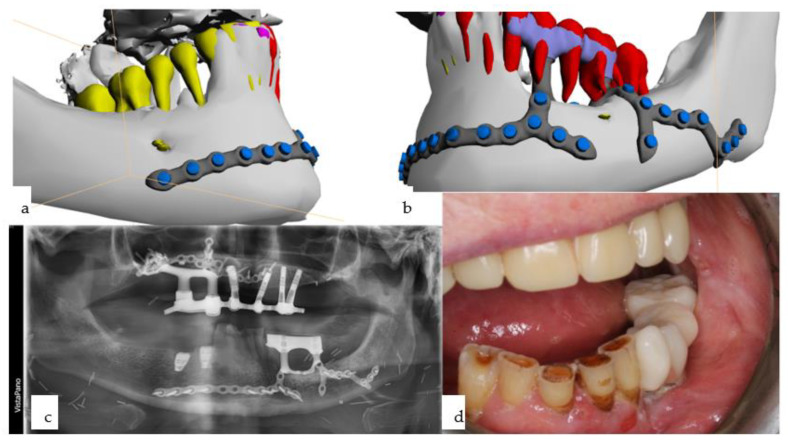
(**a**–**d**): Computer-assisted planning of the IPS Implants^®^ Preprosthetic, along with a prosthodontic backward plan superimposed (**a**,**b**). Leaving out both mental foramina is ensured; anchorage is planned far away from the transition of the posts through the radial forearm flap (**a**,**b**). The orthopantomogram shows, in addition to the aforementioned implant, two conventional bone-level implants, placed using guided drilling, in the lower right first and second premolar region (**c**). The intraoral view displays the temporary prosthesis on the mandibular subperiosteal implant.

**Figure 16:**
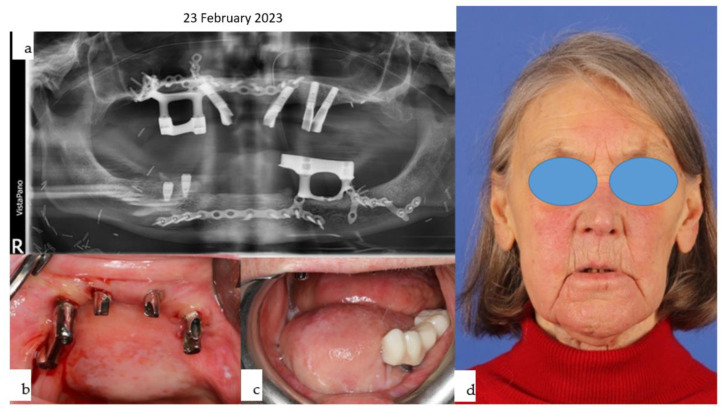
(**a**–**d**): Due to the rapid recurrence of mucosal cancer in the anterior mandible, the remaining central dentition had to be extracted along with ablative surgery for the soft tissues and marginal bone resection from the left to the right canine region (**a**). Intraoral views for the upper (**b**) and lower jaw (**c**); note: the freshly transplanted right microvascular radial forearm flap is bulging between the reconstructed floor of the mouth and the lower lip over the submerged conventional implants. The next step is to uncover the implants and provide a removable overdenture on two separate bars, i.e., left on the IPS Implants^®^ Preprosthetic and right onto the two conventional implants. Despite numerous interventions due to four ablative tumor surgeries, the patient still does not appear disfigured (**d**).

Marginal resection was performed, with alveolar bone removal posterior to the lower left canine followed by primary reconstruction using a microvascular prelaminated fasciocutaneous radial forearm flap (prelamination was provided by re-blepharoplasty of the upper eyelids), allowing separation of the horizontal (floor of the mouth) from the vertical (vestibule, lip, cheek) subunits (Figure 13 and Figure 14). Following final histopathology confirming R0 resection, a CAD/CAM subperiosteal implant was inserted 11 months later onto the left lateral mandible, with a boom running over the left mental foramen and projecting far posteriorly, while another boom was designed over the chin to the contralateral side, respecting the tooth roots (Figure 15). Multivector rigid fixation was provided by 17 2.0 screws ranging from 7 to 13 mm in length. Simultaneously, two conventional bone-level dental implants were inserted using guided surgery with a CAD/CAM drill guide designed with coDiagnostiX® 10.3 Software (Straumann, Basel, Switzerland). Twelve months later, the patient developed a fourth onset of squamous cell carcinoma of the anterior mandible, necessitating further marginal resection, including the remaining dentition of the mandible. Another microvascular radial forearm flap was used to reconstruct the intraoral tissues and separate the aforementioned subunits (Figure 16). Only the provisional prosthesis on the IPS Implants^®^ Preprosthetic, together with the implant-borne overdenture in the maxilla, allowed retention of the occlusal relationship and definition of the vertical height.

#### 2.2.7. Challenges and Adjustments

Massive soft tissue changes compared to regular anatomy made it difficult to use conventional dental implants on the right side despite individualized abutments. The originally intended timeline for manufacturing the final prosthesis had to be postponed due to disease-related therapeutic steps.

### 2.3. Case No. 3

#### 2.3.1. Patient Presentation

**Figure 17:**
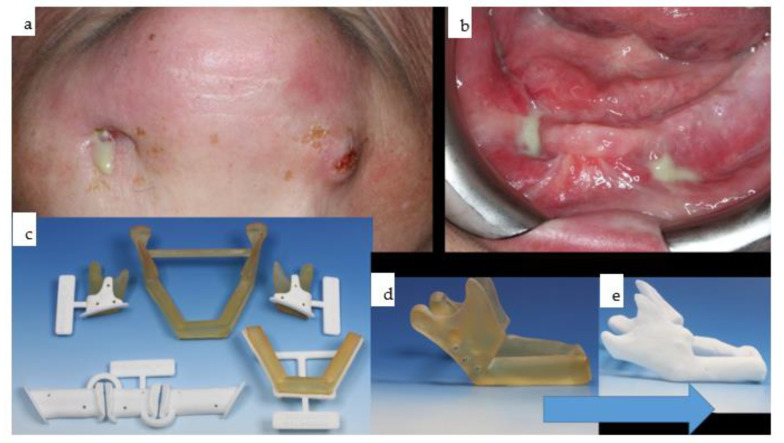
(**a**–**e**): The patient presented with extra- and intraoral fistulas (**a**,**b**) due to the extensive bilateral mandibular necrosis. Following mandibular ablation, preoperative planning for microvascular fibular osteocutaneous reconstruction was performed with multiple cutting guides for both proximal segments and the fibula, as well as a dummy for the isolated triparted fibular reconstruction. The combined and integrated neomandible attached to the remaining original bony structures is shown as separate biomodels (**c**,**d**). The intended design for the mandibular reconstruction (**d**) changed over time due to the upward rotation of the bilateral proximal articulating segments before the insertion of a subperiosteal implant was planned (**e**). A possible reason might be the loss of intermaxillary space due to the non-existent occlusion.

**Figure 18:**
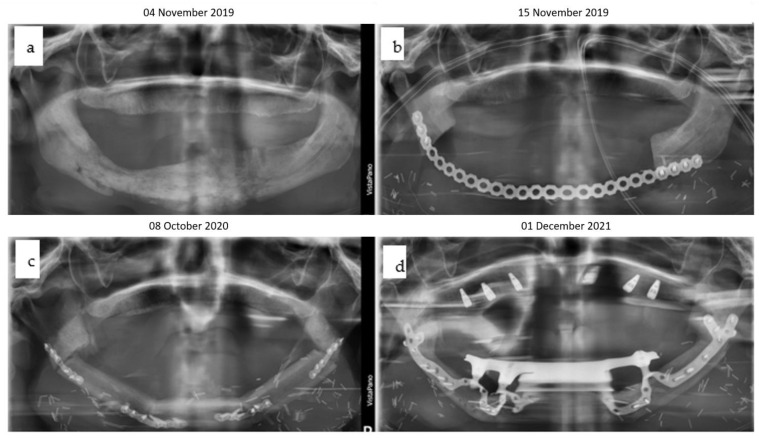
(**a**–**d**): Within two years, the patient presented with bilateral osteonecrosis of the mandible (**a**) and underwent bilateral mandibular resection with a long bridging plate (DePuys Synthes^®^, Raynham, MA, USA), along with a lateral upper arm free flap (intraoral) and a radial forearm flap (extraoral) (**b**). Eleven months later, the microvascular osteocutaneous fibular flap was inserted (**c**). Fourteen months later, the patient-specific subperiosteal implant (IPS Implants^®^ Preprosthetic) was placed in the neomandible, together with 6 conventional bone-level implants in the maxilla (**d**). A key feature of the subperiosteal implant is its fixation far away from the transition of the posts through the soft tissue.

**Figure 19:**
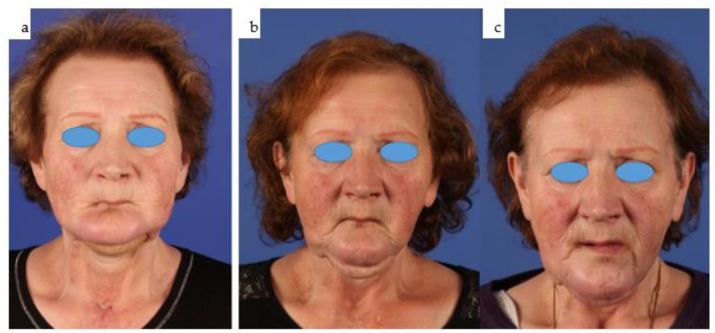
(**a**–**c**): The patient underwent primary resection and bridging of the remaining mandibular segments, along with intra- and extraoral soft tissue flap reconstruction (**a**). The second-stage surgery involved the microvascular osteocutaneous fibular flap for bony mandibular reconstruction (**b**), followed by dental rehabilitation based on six conventional dental implants in the maxilla and a customized subperiosteal implant in the mandible (**c**).

A 65-year-old female patient undergoing treatment for metastatic breast cancer with bisphosphonates developed severe osteonecrosis of the mandible (Figure 17, Figure 18 and Figure 19).

#### 2.3.2. Initial Surgery

Bilateral resection of the necrotic mandible from angle to angle was performed, followed by reconstruction using an alloplastic 2.4 bridging plate (DePuys Synthes^®^, Raynham, MA, USA) and a microvascular lateral upper arm free flap to separate the the vertical from the horizontal subunits. The broader fat cuff of the fasciocutaneous flap enveloped the bridging plate from above, while a microvascular radial forearm flap revitalized the compromised submandibular tissues and enveloped the bridging plate from below (Figure 18). Subsequent early secondary bony reconstruction was planned using computer-assisted CAD/CAM cutting and positioning guides for a microvascular osteocutaneous fibula flap, including a CAD/CAM biomodel for shaping the intended three-piece fibular bone graft (Figure 17). Fixation of the microvascular fibular graft was achieved using 2.0 miniplates.

#### 2.3.3. Challenges and Subsequent Surgery

Edentulism complicated the maintenance of the vertical relationship between the edentulous maxilla and the reconstructed mandible (Figure 18). Dental rehabilitation based on prosthodontic backward planning was performed, including a wax-up to redefine the intermaxillary vertical position. Typical upwards rotation of the bilateral ascending rami of the neomandible was observed (Figure 17), indicating that muscular and soft tissue pull alone could not maintain the post-reconstruction form without vertical occlusal stability for the 3D reconstruction of the tri-parted fibular bone graft.

#### 2.3.4. Implant Placement and Prosthodontic Suprastructures

**Figure 20:**
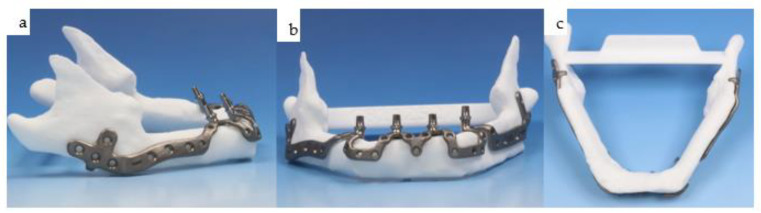
(**a**–**c**): At the time of dental rehabilitation, the IPS Implants^®^ Preprosthetic is provided along with the individual biomodel of the patient (**a**–**c**). Note: the subperiosteal implant allows for a one-fit-only approach due to its complex 3D design, minimizing the use of metal around the transition through the soft tissues.

**Figure 21:**
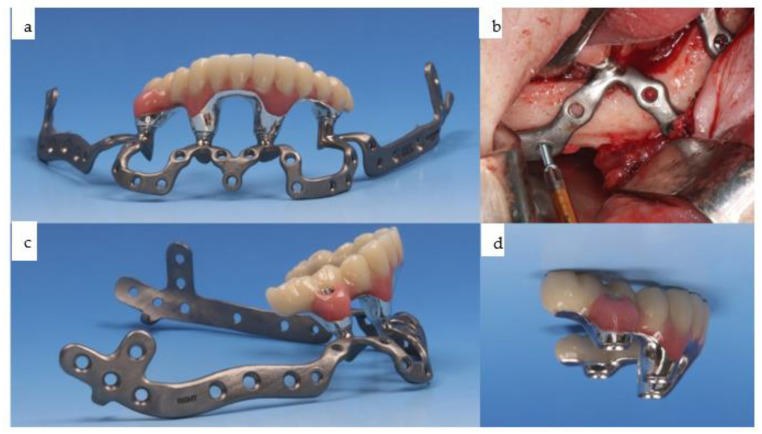
(**a**–**d**): The same IPS Implants^®^ Preprosthetic, along with the first provisional prosthesis (**d**) in a high-water design, is mounted and screw-retained (**a**,**c**,**d**), and during multivector screw fixation with 2.0 screws (**b**).

**Figure 22:**
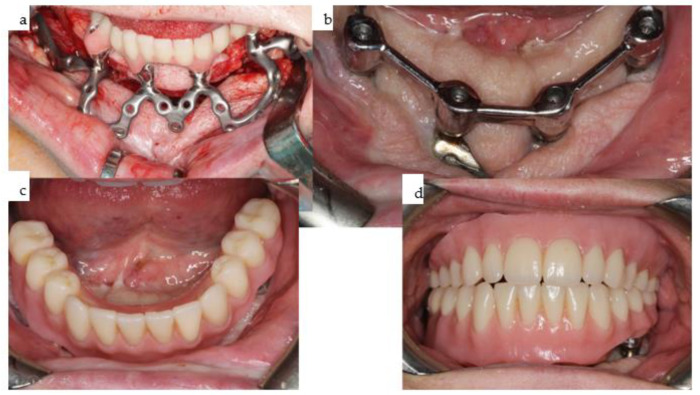
(**a**–**d**): The same IPS Implants^®^ Preprosthetic during intraoral placement (**a**), following the final prosthodontic restoration including the screw-retained non-precious metallic bar onto the IPS Implants^®^ Preprosthetic in the mandible (**b**), along with the removable overdenture on top (**c**), and in function with the upper overdenture, which was also bar-retained (**d**).

Twenty-one months post microvascular bone flap and previous soft tissue reconstruction, a subperiosteal implant containing four posts was rigidly fixated with 20 2.0 self-tapping screws, including bilateral long extensions as booms to each ascending ramus (Figure 20). The design aimed to maximize fixation at the bilateral ascending mandibular ramus area, benefiting from the well-vascularized thick soft tissue envelope provided by the pterygoid–masseteric–muscular sling. A meander-shaped design for the IPS Implants^®^ Preprosthetic was chosen to allow for secure placement on top of the fibular bone graft to allow for a secure one-fit-only shape of the footplate framework and reduce the amount of metal around the pillars passing through the overlying soft tissues (Figure 20 and Figure 21). The patient-specific subperiosteal implant was placed during outpatient surgery under general anesthesia. The first provisional superstructure for the mandibular subperiosteal implant was a high-water-designed non-precious metallic bar-based screw-retained prosthesis on four implant posts (Figure 21 and Figure 22), recommended to prevent compression to the underlying soft tissues. Six conventional bone-level tapered dental implants were placed in the maxilla guided by CAD/CAM drill guides designed with coDiagnostiX^®^ software (Straumann, Basel, Switzerland) and printed with a Formlab 3D printer (Figure 18 and Figure 22).

#### 2.3.5. Prosthodontic Plan

**Figure 23:**
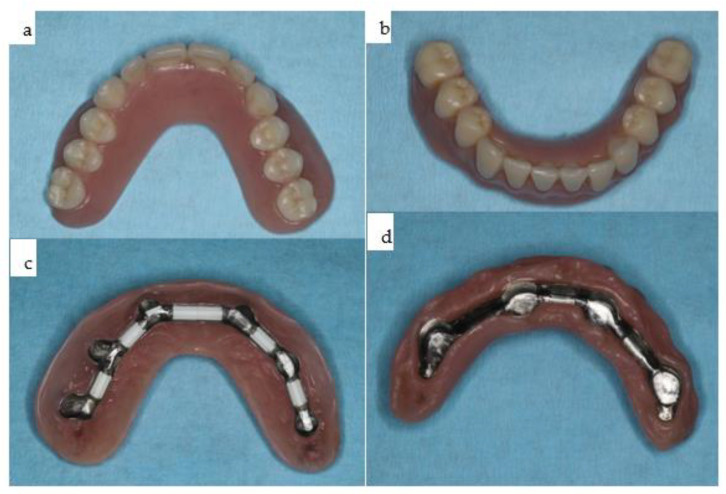
(**a**–**d**): The two overdentures for the maxilla (**a**,**c**) and the mandible (**b**,**d**).

Six conventional dental implants were chosen for the maxilla to accommodate the interaction of chewing forces and the resulting biomechanical needs, given the mobile mandible reinforced with a primarily stable rigidly fixated subperiosteal implant, interacting with a non-mobile compromised maxilla. Primarily, there were no biomechanical limitations for loading the subperiosteal implant. However, due to the need for secondary stability in the maxilla for 3.5 months before placing healing abutments onto the six conventional implants, loading in the early phase was not a key issue. A bimaxillary final overdenture was planned, with a free hard palate in the maxilla and a non-precious bar retained as a final prosthesis in the mandible (Figure 23).

#### 2.3.6. Outcome

**Figure 24:**
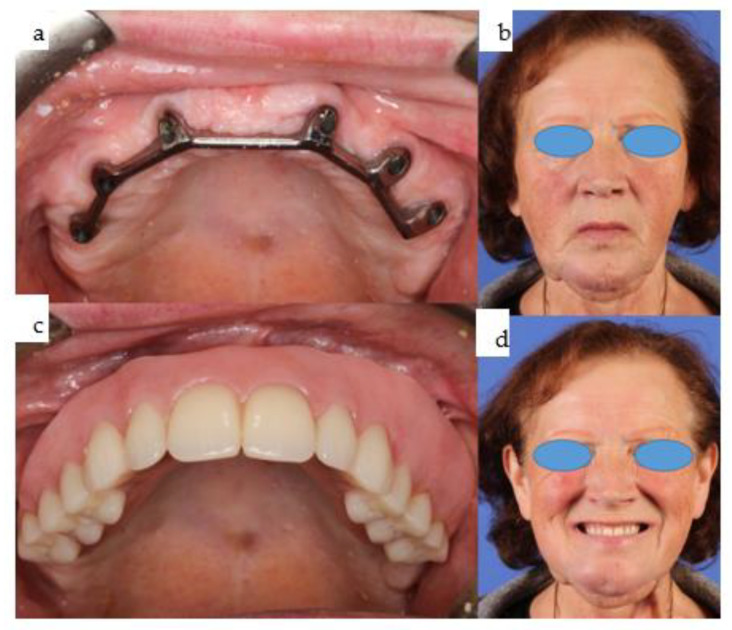
(**a**–**d**): Intraoral view of the bar mounted onto the six conventional dental implants in the maxilla (**a**), with the maxillary overdenture on top (**c**); b and d show the en face view of the patient with the incorporated upper and lower overdenture, with closed lips and smiling.

The patient has been fully functional for 18 months, able to eat without dietary or biomechanical restrictions (Figure 22, Figure 23 and Figure 24). This comprehensive treatment approach addressed the challenges of severe osteonecrosis of the mandible, facilitating successful dental rehabilitation and functional restoration for the patient.

### 2.4. Case No. 4

#### 2.4.1. Patient History

**Figure 25:**
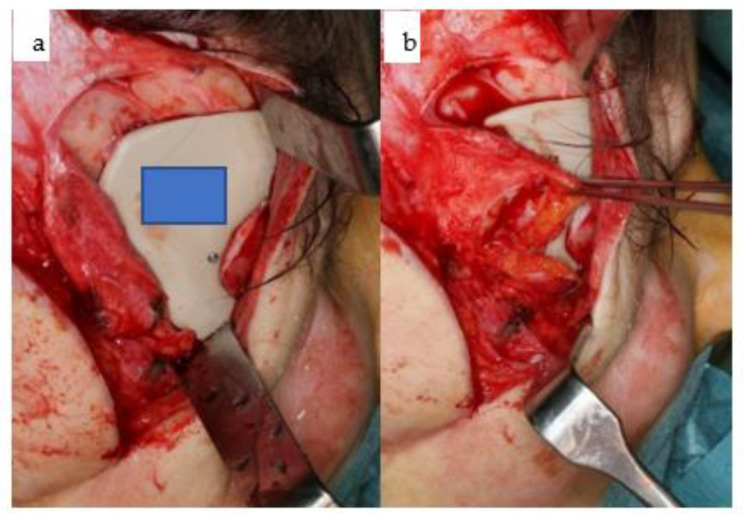
(**a**,**b**): Intraoperative clinical views of the right temporal region depicting the retraction (**a**) and replacement (**b**) of the right temporalis muscle over the inserted patient-specific PEEK implant (KLS Martin Group, Tuttlingen, Germany) to conceal the right temporal hollowing.

At the age of 3 years, a girl developed rhabdomyosarcoma of the right midface with lung metastases, necessitating radio- and chemotherapy. Severe growth disturbance and dental dysplasia resulted from the treatment. At 14 years old, she faced a squamous cell carcinoma, leading to hemiablation of the right mandible and exarticulation of the temporomandibular joint. Soft tissue reconstruction involved microvascular latissimus dorsi myocutaneous flaps, and an upper eyelid gold weight implant was placed to address facial nerve palsy. A patient-specific PEEK implant was inserted to address significant right temporal hollowing (Figure 25).

#### 2.4.2. Initial Reconstruction

**Figure 26:**
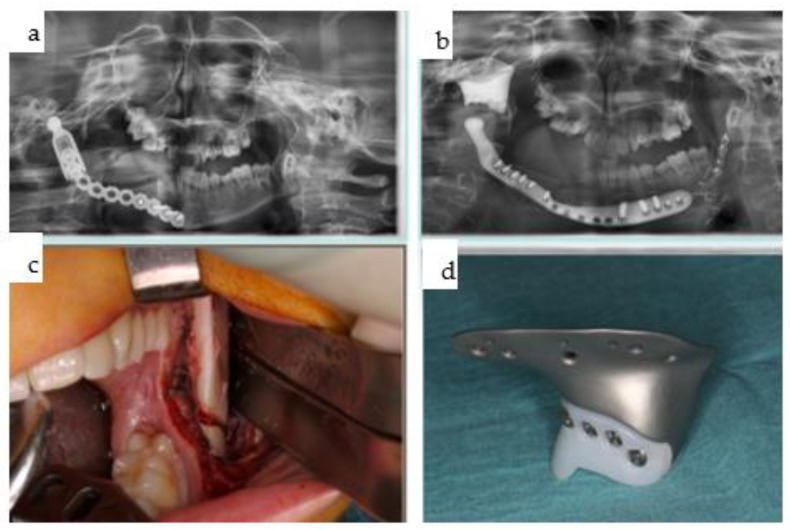
(**a**–**d**): Two orthopantomograms show the transition from the non-patient-specific 2.4 reconstruction plate with a condylar attachment without a fossa component (DePuy Synthes^®^, Raynham, MA, USA) (**a**) to a right patient-specific total joint replacement (Zimmer Biomet [Warsaw, Indiana, USA]) with a long boom serving as a contralateral mandibular implant extension for rigid fixation (**b**). Initially, bone grafting for the lateral right mandible was performed to facilitate a conventional dental implant rehabilitation protocol, which was later altered. The intraoral view (**c**) depicts a compensatory contralateral sagittal split osteotomy, while the hybrid metallic and polyethylene cranial and fossa components were documented intraoperatively prior to insertion (**d**).

**Figure 27:**
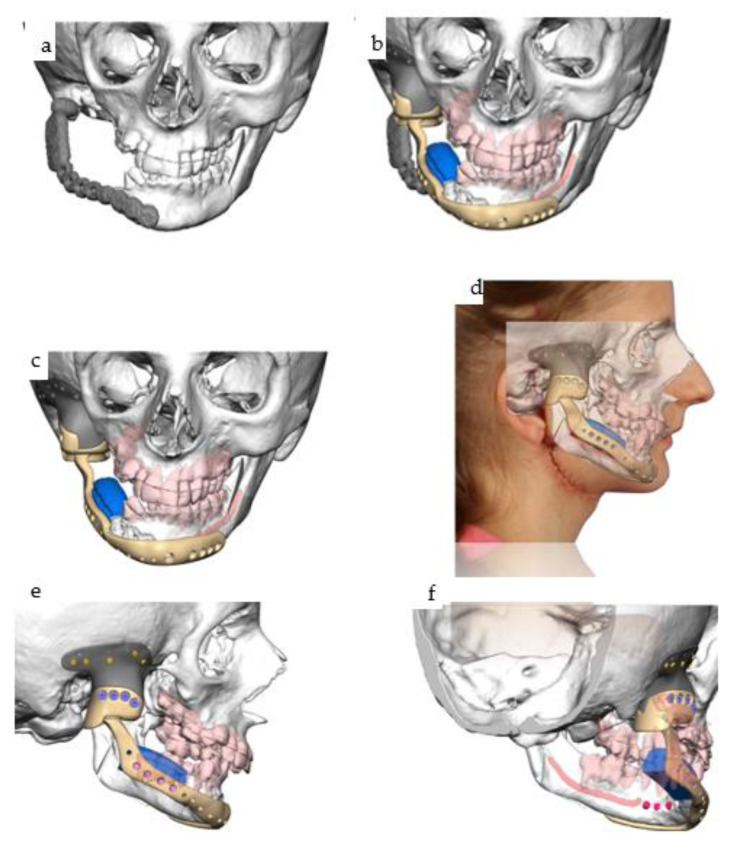
(**a**–**f**): Screenshots from the Zimmer Biomet planning platform (Warsaw, IN, USA) display the initial manually bent 2.4 reconstruction plate (see Figure 25a) with a condylar attachment (**a**), superimposed with the intended design of the Zimmer Biomet patient-specific total joint replacement, which includes the preplanned bone graft (blue), and without image fusion (**c**). Lateral right views illustrate the computer-aided design of the total joint replacement and the preplanned osteoplasty (**d**–**f**).

**Figure 28:**
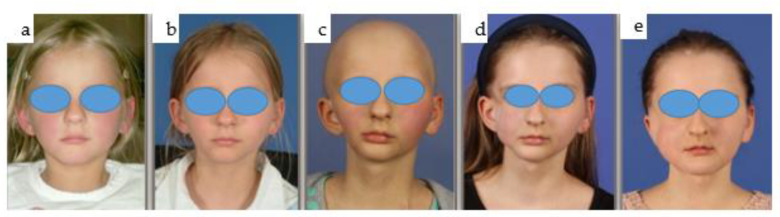
(**a**–**e**): Clinical en face views of the patient, starting from 3 years old (**a**) through young adulthood, where the growth disturbance with significant left convex facial scoliosis is evident (**b**,**c**); the mandibular deviation is corrected with the total joint replacement (**d**). Following dental rehabilitation (see below), the patient has nearly unrestricted oral function (**e**).

A 2.4 reconstruction plate was utilized along with a screw-retained condylar attachment (Figure 26, Figure 27 and Figure 28). Despite challenges, the patient maintained good oral function and mouth opening.

#### 2.4.3. Total Joint Replacement

At 19 years old, a right total joint replacement was planned to restore regular mouth opening. The fossa implant comprised a metallic base with a radiolucent articulating component made from polyethylene. The condylar component incorporated a patient-specific mandibular implant to preserve mandibular projection and rigid fixation to the remaining original mandible (Figure 25 and Figure 26).

#### 2.4.4. Challenges with Dental Rehabilitation

Edentulism in the right mandible and partially dentate right maxilla precluded achieving occlusion on the right side. Initially, free iliac crest bone grafting for the right mandible was planned to support future conventional dental implants (Figure 25 and Figure 26). Concerns arose regarding the proximity of the personalized mandibular extension of the total joint replacement to the conventional dental implants.

#### 2.4.5. New Treatment Protocol

**Figure 29:**
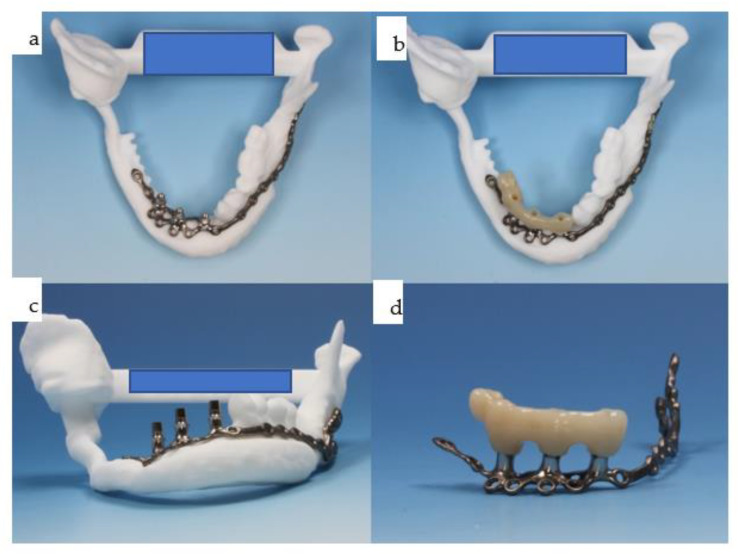
(**a**–**d**): Different views of the mandibular IPS Implants^®^ Preprosthetic mounted onto the individual biomodel (**a**–**c**) show the complex 3D geometry in a one-fit-only design, with a long boom serving as an extension to the contralateral posterior mandible. The high-water design of the first provisional prosthesis is depicted in (**d**).

**Figure 30:**
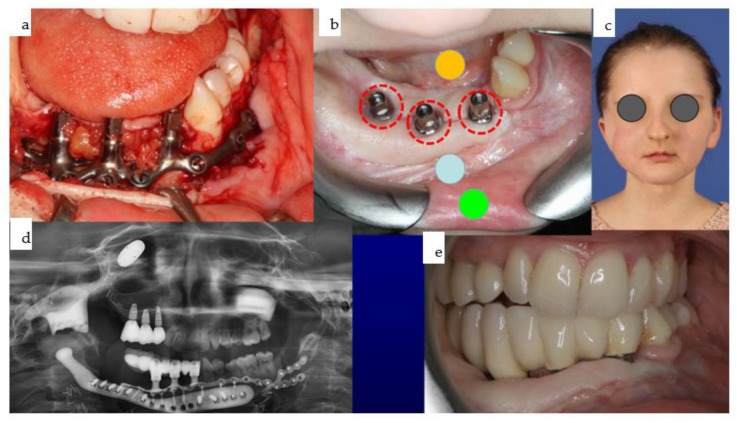
(**a**–**e**): Different views of the IPS Implants^®^ Preprosthetic during (**a**) and three months after (**b**) insertion are presented; the red dotted circles around the posts reveal healthy tissues (**b**). The yellow (floor of the mouth), light blue (vestibule), and green (inner lining of the lip) spots demonstrate the successful subunit separation due to the additional microvascular radial forearm flap, which was placed ahead of bimaxillary dental rehabilitation. The clinical appearance is depicted in (**c**); the orthopantomogram summarizes all biomaterials and implants placed into the patient over many years, including the gold weight implant for the right upper eyelid, patient-specific PEEK implant (non-radio-opaque) for the right temporal fossa (only fixation screws are visible), right total joint replacement, IPS Implants^®^ Preprosthetic with mounted final superstructure, and the conventional three bone-level tapered dental implants with crowns in the right maxilla (**d**). An intraoral view matching the clinical picture (**c**) is shown in (**e**) with the dental arches in occlusion. Mild right mandibular sagging led to occlusal correction over time.

Soft tissue transplantation with a microvascular radial forearm flap was performed to separate the right cheek from the floor of the mouth. Three months later, a CAD/CAM-manufactured IPS Implants^®^ Preprosthetic for the right lower jaw was combined with guided-surgery insertion for three conventional dental implants in the right maxilla (Figure 29 and Figure 30). Finite Element Method (FEM) analysis guided the design of the subperiosteal implant, limiting the metallic footplate around the post-carrying suprastructure and incorporating an extension as a boom to the contralateral mandible, ensuring a safe distance from the natural dentition and the patient-specific mandibular implant above the left mental nerve.

#### 2.4.6. Surgical Procedure

**Figure 31:**
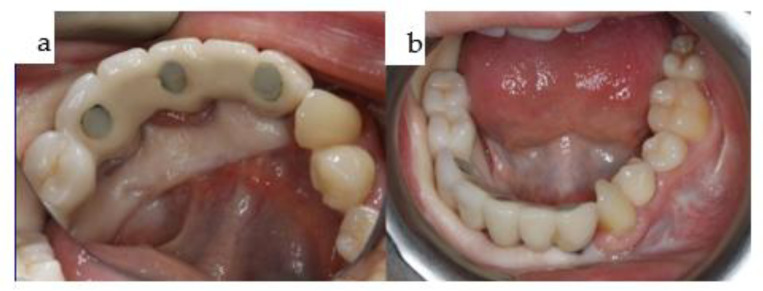
(**a**,**b**): Two intraoral views showing the final mandibular superstructure from the lingual perspective (**a**) and from the occlusal perspective (**b**).

During an outpatient procedure under general anesthesia, the subperiosteal implant was securely fixed with 18 2.0 screws, varying in length from 7–13 mm. A screw-retained provisional prosthesis with a high-water design was mounted to prevent compression of the underlying soft tissue (Figure 30). The drill guide for the guided surgery protocol was designed using coDiagnostiX^®^ software and printed in-house with autoclavable resin. Generic sleeves, narrowed to a 5 mm inner diameter with resin, were used instead of metallic sleeves. This modification, which was validated in our in-house dental lab, was employed alongside conventional Straumann reduction spoons during the ascending drilling protocol for dental implant placement. The final prosthesis was screw-retained due to inadequate interocclusal space for a removable denture (Figure 31).

#### 2.4.7. Adjustments and Outcome

Occlusal adjustments were necessary due to right mandibular sagging (Figure 30). Despite challenges, the patient experiences good function, including mouth opening, jaw motion, and chewing competence. This innovative treatment approach addressed the complex needs of the patient, providing functional restoration and enhancing quality of life despite significant medical challenges.

## 3. Discussion

Traditionally, reconstruction protocols following maxillary, midfacial, and mandibular ablation aim to replace missing tissues: soft tissue through soft tissue transfer and bony tissue through bone transfer [7].

However, our newly presented protocol deviates significantly from this approach. In our protocol, missing soft tissue subunits must be fully reconstructed for both the mandible and the maxilla. However, a partially or fully resected maxilla does not necessitate bone grafting. This distinction arises from the intrinsic nature of the maxilla and midface as integral components of the craniofacial skeleton. Consequently, a one-piece implant in the maxilla can conform to a rigid, albeit irregular, surface and withstand resulting biomechanical forces. Thus, even following total maxillectomy, no bone grafting is required for the maxilla. Instead of inserting a bone graft between the remaining original maxillary and midface structures—necessitating significant mini-plates for fixation—the length and vector of the pillars of IPS Implants^®^ Preprosthetic can be tailored to meet prosthodontic requirements precisely. However, adequate soft tissues are essential before IPS Implants^®^ Preprosthetic insertion to accommodate subsequent contour and projection changes due to either temporary or final superstructure placement [8].

The view of the maxilla and midface differs notably from that of the mandible. Unlike the maxilla, the mandible is an entirely independent bone interacting with the skull solely through fixation and movement facilitated by muscles, fibrous tissues, vessels, nerves, and skin. The position of the mandible is defined in relation to the skull by the bilateral condyle-to-fossa relationship and occlusion between the upper and lower dental arches. Therefore, any segmental resection of the mandible necessitates primary bone grafting, unlike the maxilla, to biomechanically unify the bilaterally independent temporomandibular joint subunits [2,9]. Alternatively, in cases of hemimandibular ablation, including exarticulation, patient-specific total joint replacement in CAD/CAM technology serves as a reliable method to maintain the form and function of the lower face by accurately reconstructing the correct posterior height and sagittal projection of the remaining mandibular hard tissues.

Complications associated with the IPS Implants^®^ Preprosthetic may arise from insufficient soft tissue coverage and the absence of separated anatomical subunits, particularly in the mandible, where the vertical subunit (cheek, lip, vestibule) functions independently from the horizontal subunit (floor of mouth, tongue). To mitigate this, robust vascularized soft tissue transplantation must be considered as the initial reconstruction step before IPS Implants^®^ Preprosthetic placement. This potential complication parallels those encountered in conventional dental implant treatment, although framework exposure does not necessarily result in implant loss with the IPS Implants^®^ Preprosthetic. Fracture of the IPS Implants^®^ Preprosthetic has not been observed thus far.

While the question of bone reconstruction seems to follow a predictable protocol, there is a clear risk of non-forgiving situations when it comes to soft tissue requirements, i.e., not only must the amount of tissue loss be addressed with the soft tissue assessment, but the function and interaction of horizontally and vertically neighboring anatomical subunits must also be assessed [10]. In addition to this, apart from tissue loss with missing subunits, a potential further significant limitation might result from adjuvant therapy (e.g., irradiation, chemo-, immunotherapy) [11]. In such cases, the vascularized envelope may even have to be overcorrected—mainly with microvascular soft tissue flaps—so that the separation of subunits is achieved to avoid negative effects like scarring, shrinkage, etc. This selective soft tissue protocol has to be followed both in oncology patients and post-traumatic tissue loss. However, it does not need to be applied this strictly either in atrophic cases or in the majority of cleft patients.

Another traditional rule for maxillary and mandibular reconstruction is to plan and re-establish the vascularized bone where dental implants shall be positioned [12,13,14]. In addition to the vector limitations of a straight dental implant—although individualized abutments are used—towards prosthodontic needs to compensate significant skeletal mismatches, the presented method describes a reliable reconstruction protocol, where there is literally no limitation neither for the vector nor for the biomechanical loading [6,7]. This is guaranteed by a rigidly fixated one-piece implant carrying the required number of four posts for complete dental rehabilitation of an edentulous jaw. Apart from the vector freedom in the design of the posts, there have not been any restrictions identified in pillar length yet. No secondary alignment of the pillars has to be addressed in case of a full dental rehabilitation, where all pillars are digitally aligned parallel from the start.

In addition, to even assure the parallel position of the posts during and after treatment with regard to µm accuracy, the authors developed, together with a specialized dental lab in digital technologies (Zahntechnisches Labor Duen GmbH, Hamburg, Germany), a first provisional prosthesis that includes multiple functions: firstly, for quality control during surgery, an individual precision-made non-precious metal bar is used; secondly, for the function of a provisional prosthesis, the previously mentioned bar is mounted with acrylic teeth. Thirdly, the two-component-based provisional suprastructure is screw-retained with a prosthodontic screw intraoperatively onto the telescoping posts of the one-piece patient-specific maxillary implant, which helps to insert the subperiosteal implant and to facilitate the procedure of rigid screw fixation. Fourthly, the resistance-free installation and removal of the provisional suprastructure now proves that the correct (parallel) pillar position is maintained. Fifthly, this treatment element ensures that the whole workflow—from planning the IPS Implants^®^ Preprosthetic up to the first provisional and even the final prosthesis—could be digitally performed from the standpoint of precision. Furthermore, this non-precious-metal bar-based quality control element gains even more importance when it comes to more extended frameworks such as individual footplates with a higher number of screws and potential deformation during placement and rigid fixation. In conventional dental implant treatment, potential inaccuracy is much more likely due to the biological dependency of gaining secondary stability after individual drilling of each dental implant socket. Only the cooperation between surgeons and dental technicians made this breakthrough possible for a modern new-generation CAD/CAM subperiosteal implant, when the individualized suprastructure also became part of the digital workflow, meeting the high accuracy requirements in implant dentistry.

Concerning the footplate design, an individual design should include additional 3D applications contributing to a one-fit-only design. For the maxilla, little hooks should be placed at the piriform aperture and the malar prominence should be embraced from below. The latter facilitates transoral drilling and screw insertion as well as stability onto strong bone. The typical buttresses of the midface should be used for anchorage, including the subnasal midline. For the mandible, the mental nerves should be left out and, if possible, the boom of the footplate extending posteriorly should be designed above the nerve in case of transoral placement of the customized subperiosteal implant.

Further design key points exist for the IPS Implants^®^ Preprosthetic that proved to be of value:

Use as little metal as possible (according to the individual FEM analysis) around and close to the transition zone of the posts through the soft tissue and on the crestal bone.

Use a one-piece implant design per jaw.

Use a complex 3D design to catch even irregular recipient sites in a one-fit-only design match (including small arms as checkmarks at the piriform aperture).

Use an extension of the framework far away from the transition zone through soft tissue and levering onto valid and vascularized strong bones.

Use the variable thickness of the footplate according to biomechanical requirements.

Use a sloped design at the end of each boom of the footplate.

Use four posts to sufficiently dentally rehabilitate an edentulous jaw.

Use multivector rigid screw fixation.

All mentioned aspects contribute to a biomechanical loading equivalent to normal chewing forces. The literature shows that many of the above-mentioned technical hints are not considered, despite currently increasing enthusiasm about subperiosteal implants for dental rehabilitation [15,16,17,18]. Apart from the design features, rigid and far-away fixation from the transition zone of the posts through the soft tissue into the oral cavity is not shown in the literature either [19,20]. Many cases are published in which the amount of metal close to the transition zone through the soft tissue is too high, the extension of the footplate is too small, the number of screws is too low and the position of the screws is too close to the posts [21,22,23,24,25,26,27,28,29]. Evaluation of patient satisfaction after dental rehabilitation using modern sub-periosteal CAD/CAM implants in severe cases shows that this less invasive technique has a high acceptance [30,31].

Like any other technique, the use of the IPS Implants^®^ Preprosthetic has its limitations. For instance, the planning process is more time-consuming and requires expertise, particularly when it comes to judging the quality and functionality of the surrounding soft tissues. This aspect must be scrutinized even more critically if comorbidities, general health restrictions, or medical treatment-related compromises exist (such as immunotherapy, radiotherapy, chemotherapy, or antiresorptive drugs).

## 4. Conclusions

The presented treatment protocol fundamentally differs from historical subperiosteal implants. The IPS Implants^®^ Preprosthetic constitutes a modern extension in implant dentistry, offering a personalized solution for dental rehabilitation of the maxilla and/or mandible in exceptional cases. This is particularly applicable when conventional dental implant protocols have failed or would necessitate excessively invasive measures, which are no longer acceptable to patients. Additionally, traditional dental rehabilitation strategies are often too time-consuming for elderly or medically compromised patients, such as those undergoing oncological treatment.

The design of the IPS Implants^®^ Preprosthetic is highly functionalized and preventive, aiming to minimize adverse effects. Its multivector screw retention allows for primary stability and direct loading. The entire process, from planning to manufacturing up to the final prosthesis, is conducted in a fully digital workflow. It has become common practice to combine the IPS Implants^®^ Preprosthetic with conventional dental implant treatment, either in the same jaw or the opposite jaw, either as a primary or secondary option.

## Data Availability

The raw data supporting the conclusions of this article will be made available by the authors on request.
